# Stereo-Selectivity of Human Serum Albumin to Enantiomeric and Isoelectronic Pollutants Dissected by Spectroscopy, Calorimetry and Bioinformatics

**DOI:** 10.1371/journal.pone.0026186

**Published:** 2011-11-02

**Authors:** Ejaz Ahmad, Gulam Rabbani, Nida Zaidi, Saurabh Singh, Mohd Rehan, Mohd Moin Khan, Shah Kamranur Rahman, Zainuddin Quadri, Mohd. Shadab, Mohd Tashfeen Ashraf, Naidu Subbarao, Rajiv Bhat, Rizwan Hasan Khan

**Affiliations:** 1 Interdisciplinary Biotechnology Unit, Aligarh Muslim University, Aligarh, India; 2 School of Biotechnology, Jawaharlal Nehru University, New Delhi, India; 3 School of Computational and Integrative Sciences, Jawaharlal Nehru University, New Delhi, India; 4 School of Biotechnology, Gautam Buddha University, Greater Noida, India; German Cancer Research Center, Germany

## Abstract

1–naphthol (1N), 2–naphthol (2N) and 8–quinolinol (8H) are general water pollutants. 1N and 2N are the configurational enantiomers and 8H is isoelectronic to 1N and 2N. These pollutants when ingested are transported in the blood by proteins like human serum albumin (HSA). Binding of these pollutants to HSA has been explored to elucidate the specific selectivity of molecular recognition by this multiligand binding protein. The association constants (K_b_) of these pollutants to HSA were moderate (10^4^–10^5^ M^−1^). The proximity of the ligands to HSA is also revealed by their average binding distance, r, which is estimated to be in the range of 4.39–5.37 nm. The binding free energy (ΔG) in each case remains effectively the same for each site because of enthalpy–entropy compensation (EEC). The difference observed between ΔC_p_^exp^ and ΔC_p_^calc^ are suggested to be caused by binding–induced flexibility changes in the HSA. Efforts are also made to elaborate the differences observed in binding isotherms obtained through multiple approaches of calorimetry, spectroscopy and bioinformatics. We suggest that difference in dissociation constants of pollutants by calorimetry, spectroscopic and computational approaches could correspond to occurrence of different set of populations of pollutants having different molecular characteristics in ground state and excited state. Furthermore, our observation of enhanced binding of pollutants (2N and 8H) in the presence of hemin signifies that ligands like hemin may enhance the storage period of these pollutants in blood that may even facilitate the ill effects of these pollutants.

## Introduction

α–naphthol [1–naphthol (1N)], β–naphthol [2–naphthol (2N)] and 8–quinolinol [8–hydoxy quinoline (8H)] are non-persistent organic water pollutants. They have similar properties. They are sparingly soluble in water and exhibit antiseptic properties. One way in which naphthols differ from each other is the form of their crystals. 1N crystallizes in prisms and 2N in plates. Cellular presence of 1N causes depolymerization of spindle microtubules and apparent uncoupling of karyokinesis and cytokinesis in mitotic cells. Although at a much lower frequency, presence of 2N also causes similar configurations. 1N has been investigated as a mutagen and reproductive effector and has also been associated with reduced testosterone levels [1]. Naphthols are used in the synthesis of certain azo–dyes and antioxidants for rubbers and as indicators in chemical analyses. 8H is used as a metal chelating agent, in preparing antiseptics, deodorants, fungicides etc. Their accumulation in body causes cyanosis, liver damage, nephritis, circulatory collapse and even death.

All these three pollutants are transported in blood by plasma proteins like human serum albumin (HSA). HSA is a 585 amino acid long heart-shape monomer comprising three structurally homologous domains each of which displays specific and functional characteristics. Each of these three domains is composed of sub-domains A and B providing flexibility to the protein molecule so that the protein can bind to a variety of ligands. HSA is responsible for the transport, storage and metabolism of many therapeutic drugs in the blood thereby restricting their free, active concentrations and therefore can significantly affect their pharmacokinetics and metabolism. Two distinct binding sites, commonly referred to as Sudlow site 1 and site 2, have been identified in HSA for various drugs [2]. Site 1 binds to bulky hydrophobic and heterocyclic molecules with a centrally located negative charge (e.g. warfarin, phenylbutazone). Site 2 binds to aromatic carboxylic acids with a negative charge at one end distal from the remaining hydrophobic structure (e.g. diazepam, ibuprofen). Binding of ligands to albumin alters the pattern and volume of distribution, lowers the rate of clearance, and increases the plasma half-life of the ligand. A detailed characterization of the protein's binding property to different ligands is therefore necessary not only to understand its key physiological functions but also to understand its impact on ligand transport and delivery.

Here we have examined the protein-ligand associations of HSA to the pollutants (1N, 2N and 8H) by utilizing spectroscopic techniques, calorimetry and computational method of molecular docking. With 1N and 2N being the configurational enantiomers with a difference in the position of –OH group and 8H being isoelectronic to 1N and 2N (–CH– of 1N is replaced by –N– in 8H), the objective of this study is to elucidate the specific selectivity of molecular recognition by a multiligand binding protein. Moreover, the differences obtained in the molecular-interaction data by multiple techniques have been dissected to understand and explore intricacies of protein-ligand interaction.

## Materials and Methods

### Materials

Essentially fatty acid free human serum albumin (A1887), 1N (N2780) and 2N (185507) were product of Sigma-Aldrich, USA whereas 8H (24874) was procured from Qualigens, India. All other reagents and buffer compounds used were of analytical grade.

### Preparation of solutions

All experiments were carried out in 20 mM Tris–HCl pH 7.4 buffer. HSA was used without further purification as its purity was checked by SDS–PAGE at high concentration. HSA was dialyzed properly against respective buffer. Its concentration was determined spectrophotometerically by using 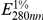 = 5.3. Pollutant stocks were stored in dark to minimize photolytic degradation.

### UV-Visible Spectroscopic measurements

Absorption measurements were performed at 37°C on Perkin-Elmer Lambda 25 double beam UV–Vis spectrophotometer attached with peltier temperature programmer-1 (PTP–1). A fixed concentration of HSA (12 µM) with increasing concentrations of pollutants from 0 to 600 µM (molar ratio of P∶L = 1∶50) were added and the pollutant blank of equal concentrations were subtracted to the protein-pollutant spectra. From these data, we can determine the dissociation constant (K_d_) for the HSA–pollutant interaction according to the following Equation [3]:
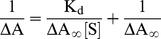
(1)where ΔA = A-A_o_ (for 1N and 2N) and ΔA = A_o_-A (for 8H); A and A_o_ were the absorption of HSA at 280 nm in presence and absence of pollutants respectively; [S] is the concentration of these pollutants; ΔA∞ is the change in the absorption where protein was completely saturated by the ligands. The association constants (K_b_) were derived from the inverse of dissociation constant (K_d_). The degree of cooperativity (*h*) for the HSA–pollutant binding systems were obtained from the Hill Equation:
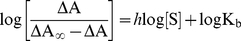
(2)in which the intercept (logK_b_) and the value of ΔA_∞_ were calculated and used from the above Equation 1. The change in free energy was calculated from:
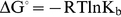
(3)where R (1.987 cal.mol^−1^.K^−1^) is the gas constant.

### Steady state fluorescence quenching measurements

Schimadzu 5301PC fluorescence spectrophotometer equipped with a constant temperature holder and the temperatures (25, 37 and 45°C) were maintained by a constant temperature water circulator (Julabo Eyela). The excitation and emission slits were both set at 3 and 5 nm respectively. The titration of the pollutants (0–100 µM) to 2 µM HSA solution was carried out in a dual-path length fluorescence cuvette (10×3.5 mm). The shorter path length was oriented towards the emission side. Such a low concentration of HSA (2 µM) with absorbance value of ∼0.07 was used throughout the fluorescence experiments to minimize the inner filter effect. Intrinsic fluorescence was measured by exciting at 295 nm to probe exclusively the tryptophan only. The emission spectra were recorded in the range of 300–500 nm and the data were plotted at 320 nm because no emission of pollutants occurred at 320 nm if excited at 295 nm. The decrease in fluorescence intensity at 320 nm was analyzed according to the Stern–Volmer Equation [4]:
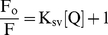
(4)where F_o_ and F were the fluorescence intensities in absence and presence of quencher (pollutants), K_sv_ is the Stern–Volmer quenching constant and:
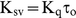
(5)where K_q_ is the bimolecular rate constant of the quenching reaction and τ_0_ the average integral fluorescence life time of tryptophan which is ∼4.31×10^−9^ sec. Binding constants and binding sites were obtained from [5]:
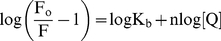
(6)where, K_b_ is the binding constant and n is number of binding sites. The change in free energy was calculated from Equation 3 whereas change in enthalpy and entropy at different temperatures were analyzed from van't Hoff Equation:
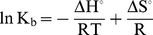
(7)and second law of thermodynamics:
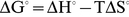
(8)where ΔG° is free energy change, ΔH° is the enthalpy change, ΔS° is entropy change.

For the determination of binding sites, titration of pollutants to HSA in absence and presence of site markers (probes) were performed as a competitive experiment. 2 µM HSA was incubated with 4 µM site markers (1∶2) to saturate completely the corresponding sites. Pollutants were gradually added to the HSA–site markers. The data were analyzed in the same way as discussed above.

### Tryptophan fluorescence resonance energy transfer (FRET) to the pollutants

The fluorescence spectra of HSA (2 µM) and absorption spectra of pollutants (2 µM) between 300 to 400 nm were scanned in similar way as given in method sections ‘Fluorescence Quenching’ and ‘UV-Visible’ experiments at 37°C. If the emission spectrum of donor (W214 of HSA) significantly overlap with the absorption spectrum of acceptor (1N, 2N and 8H), these donor-acceptor pairs will considered in Förster distance and then we could ascertain the possibility of energy transfer [6]. Therefore, the degree of energy transfer depends upon the area of overlap and the distance between these donor-acceptor molecules. The efficiency of energy transfer (E) is calculated using the following Equation [7]:
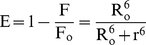
(9)where F_o_ and F were the fluorescence intensities of HSA in absence and presence of pollutants respectively; r is the distance between donor and acceptor and R_o_ is the critical distance at which transfer efficiency equals to 50% which can be calculated from the following Equation:
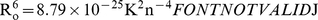
(10)where *K^2^* is the orientation factor related to the geometry of the donor and acceptor of dipoles, *n* is the refractive index of the medium, ϕ is the fluorescence quantum yield of the donor in absence of acceptor; and *J* expresses the degree of spectral overlap between the donor emission and the acceptor absorption which can be evaluated by integrating the overlap spectral area in between 300 to 400 nm from following Equation:
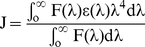
(11)where F(λ) is the fluorescence intensity of the donor at wavelength range λ which is dimensionless, and ε(λ) is the molar absorptivity (extinction coefficient) of the acceptor at wavelength λ in M^−1^ cm^−1^. In our present study *K* ^2^, ϕ and *n* were taken as 2/3, 0.118 and 1.336 respectively [8].

### Isothermal titration calorimetry

The calorimetric measurements were carried out on a titration calorimeter from Microcal (Northampton, MA) at 15, 25, 37 and 45°C in 20 mM Tris–HCl buffer pH 7.4. The solutions of HSA and pollutants were filtered and degassed properly on Thermovac immediately before titrations. To fill the cell, the titrand was filled in a syringe and the syringe was tapped gently with the needle pointing upward and slightly working the plunger up and down to remove any trapped air bubble in the syringe. Now, the solution of 24.4 µM HSA in the 1.44 ml sample cell was titrated with 3 mM pollutants (1N, 2N and 8H) using a 288 µl automatic rotating syringe stirring at 307 rpm. Titration experiments consisted of 36 injections of 8 µl each of duration 20 s with 2 s filter period and 180 s spacing between each injection. The analog input range was +/−1.25 V and the reference power was set at 20 µcal s^−1^. The heat associated with each injection was observed as a peak that corresponded to the power required to keep the sample and reference cells at identical temperatures. Control experiments were performed by titrating pollutants into the same buffer to obtain the heats of ligand dilution. The net enthalpy for each HSA–pollutant association was determined by subtraction of the component heats of dilution from each injection heat pulse. Integration with respect to time of the heats produced per serial injection of pollutant yielded the corresponding binding isotherm. The binding isotherms were best fitted for sequential binding sites [9] by using Origin 7.0 provided with the MicroCal instrument. The least *χ* ^2^ values or lowest errors were obtained to a two site sequential binding model from Marquardt minimization algorithm [10] to obtain best fitting values until constant *χ*2 values were achieved for determination of the association constants (K_b_ values), stoichiometry (n) and enthalpy change (ΔH). Other thermodynamic parameters such as change in free energy (ΔG) and change in entropy (ΔS) were obtained from Equations 3 and 8. Please go through **Supplementary Materials S1**. The temperature dependence of enthalpy change of molecular association contributes to the change in specific heat capacity [11]:
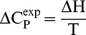
(12)


### Molecular Docking Studies

The PDB structure 1AO6 of HSA was taken for molecular docking of phenolic compounds 1N, 2N and 8H to site 1 and site 2 of HSA. The complexes of HSA with site 1 markers (Warfarin, 1H9Z; Phenylbutazone, 2BXC) and with site 2 markers (Diazepam, 2BXF; Ibuprofen, 2BXG) were downloaded from Brookhaven Protein Databank. The residues falling within 5 Å of the above sites were extracted and combined to define the binding site residues. From Pubchem database the SDF format for 3D structures of 1N (CID: 7005), 2N (CID: 8663) and 8H (CID: 1923) were downloaded. Molecular docking simulations of all the three phenolic compounds were performed with Autodock4.0 program [12]. Autodock uses Lamarkian genetic algorithm to calculate the possible conformations of the ligand that binds to the protein. Gasteiger charges were added to the ligands. Polar hydrogen atoms, Kollman charges were merged to the protein. A Grid of 60×60×60 Å with spacing of 0.375 Å was generated, covering all the active site residues. For docking simulations the parameters were set to 10 GA runs terminating after a maximum of 25, 00,000 energy evaluations, population size was set to150 and crossover rate of 0.8. For flexible docking, the above parameters were same, but the residues involve in rigid docking as well as some other important neighboring residues of binding sites were set to flexible. For further analysis, the conformer with the lowest binding energy with best fitness score was used. The binding energies of docked molecules were also calculated using X-score [13] to cross check the values obtained from Autodock4.0 program. The hydrogen bonding and hydrophobic interactions between ligand and protein were calculated using LigPlot [14]. PyMol version 0.99 [15] and chimera version 1.3 [16] were used for visualization. Differences in accessible surface area (ASA) of protein before and after ligand complexations were calculated from NACCESS version 2.1.1 [17]. The change in ASA for residue, **X** was calculated from the Equation:
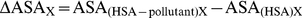
(13)Upon pollutant interaction if a residue lost more than 10 Å^2^ of its ASA, it was considered as being involved in the interaction [18].

The ITC obtained 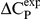, values were further compared with the calculated values (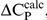) from the change in non-polar and polar accessible surface area (ΔASA) of HSA upon interaction with pollutants [19]:
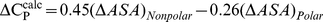
(14)


## Results

### UV-visible absorption spectroscopy

**Figure 1:**
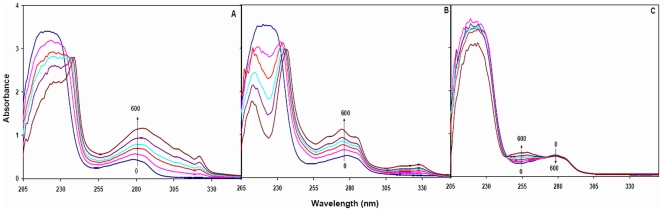
Absorption spectra of HSA gradually titrated with pollutants (A) 1N, (B) 2N, and (C) 8H at 37°C. HSA–pollutant spectra were subtracted from the spectra of equal amount of pollutants. HSA = 12 µM and 1N = 2N = 8H = 0 to 600 µM.

**Figure 2:**
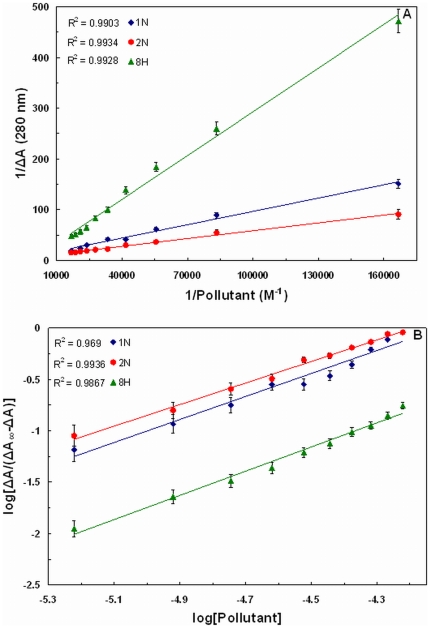
(A) Plot of 1/ΔA (at 280 nm) against 1/[S] and (B) Hill plot of log [ΔA/(ΔA∞-ΔA)] *vs* log [Pollutant] at 37°C. HSA = 12 µM and1N = 2N = 8H = 0 to 60 µM.

In UV–absorption spectrum, the far–UV region corresponds to secondary structures whereas near–UV region is related to the tertiary structures of the protein. Hence, change in absorption of protein in UV region can be used to investigate ligand induced alterations in protein and to estimate the extent of protein-ligand interaction. The UV spectral changes as observed upon pollutant titration to HSA are shown in Figure 1. Upon interaction with each pollutant the intensity of first peak at 220 nm was found to decrease gradually. Presence of 1N at higher concentration leads to formation of two new distinguishable peaks. Presence of 2N leads to formation of two clear independent peaks (at 215 and 238 nm) even at lower concentrations. Presence of 8H however was totally ineffective in generating new peaks. The intensity of HSA spectrum in near–UV region was increased with a slight red shift in the presence of 1N and 2N. Unlike these two compounds, presence of 8H decreased the intensity in the region around 280 nm and increased the intensity in the region from 244 to 274 nm with a clear observation of two isobestic points. Such type of changes in far– and near–UV regions implied that both the tertiary and secondary structures of HSA were altered upon interaction with pollutants. Dissociation constants (K_d_) as obtained from the slope of plot 1/ΔA *vs* 1/[S] for HSA–1N, HSA–2N and HSA–8H systems (Figure 2A) were estimated to be 9.66×10^−5^ M, 6.69×10^−5^ M and 50×10^−5^ M respectively. Binding constants (K_b_) for the above systems were calculated from the obtained values of K_d_ (Table 2). The observed values of ΔA∞ for HSA–1N, HSA–2N and HSA–8H systems were 0.10738, 0.13399 and 0.1727 respectively which indicated that the highest equivalence is achieved at the molar ratios of 1∶8 for HSA to 1N, 1∶11 for HSA to 2N and 1∶40 for HSA to 8H (Table 2). The slope of log [ΔA/(ΔA∞-ΔA)] *vs* log [S] plot gives the value of Hill coefficient (*h*) as a measure of or degree of cooperativity (Figure 2B) in which ΔA∞ was obtained from Figure 2A. For binding of a ligand at more than one site to a protein, the coefficient can also be deduced as the minimum number of interacting binding sites for ligands. The *h* values approaching to unity implies that HSA–pollutant interactions were non–cooperative.

### Fluorescence quenching measurements

**Figure 3:**
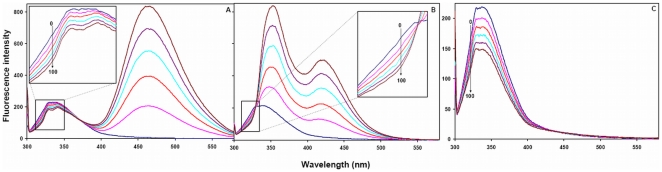
Fluorescence quenching of HSA by (A) 1N, (B) 2N and (C) 8H at 37°C. [HSA = 2 µM; 1N = 2N = 8H = 0–100 µM].

**Figure 4:**
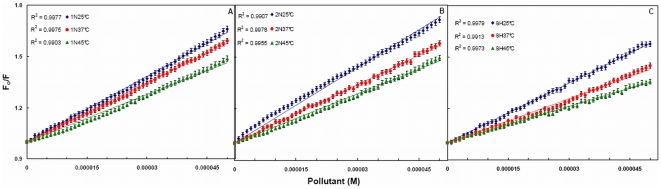
Stern-Volmer plot between Fo/F and [Pollutants] for HSA–pollutant interaction. (A) for HSA–1N, (B) for HSA–2N and (C) for HSA–8H at 25, 37 and 45°C. [HSA = 2 µM; 1N = 2N = 8H = 0–50 µM].

Quenching of HSA intrinsic fluorescence in the presence of pollutants was investigated to measure the extent of pollutant binding. Figure 3 shows that addition of the pollutants leads to a dramatic change in the emission spectra of HSA. The isobestic points of 1N titration at 373 nm and of 2N titration at 329 nm titration to HSA signify the boundaries for the contribution of HSA and pollutants in the fluorescence emission spectra. However in case of 8H there was no observation of any isobestic point as 8H does not emit in the range of 300–580 nm. As the fluorescence intensity at 340 nm (λ_max_ of HSA) overlaps with the emission of 1N and 2N, the fluorescence quenching effects of pollutants were estimated at 320 nm from the Stern–Volmer plot (Figure 4). There is a linear dependence between F_o_/F and concentration of the pollutants. It is further observed that as the temperature increases from 25°C to 45°C there was a decrease in extent of fluorescence quenching. This indicates that the temperature–induced changes in microenvironment of the protein affected the mode and mechanism of quenching and therefore HSA–pollutant complexation. Hence, the decrease in slopes with increase in temperature and as a result the decrease in K_sv_ (Table 3) signifies the static quenching mode and formation of HSA–pollutant complex. Moreover, the K_q_ values in each case of pollutants is in the range of 10^12^ M^−1^ s^−1^ (Table 3) that is 100 times greater than the maximum value for dynamic quenching, 2×10^10^ M^−1^ s^−1^ [20].

**Figure 5:**
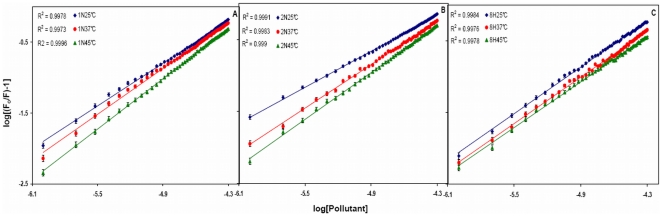
Plot between log [(F_o_/F)-1] and log[Pollutant] for HSA–pollutant interaction. (A) for HSA–1N, (B for HSA–2N) and (C) for HSA–8H at 25, 37 and 45°C. [HSA = 2 µM; 1N = 2N = 8H = 0–50 µM].

To determine the binding constant and number of binding sites log[(F_o_/F) −1] *vs* log[Pollutant] is plotted (Figure 5). The slope of the plot provides number of binding sites (*n*). The binding constant (K_b_) of the ligand can be calculated from the intercept of this plot (Equation 6). Single binding site (*n*) is obtained for all the three pollutants. Due to the presence of only one tryptophan residue (W214), the qualitative homogeneity in emitted fluorescence is clearly shown by non–deviation from linearity in the quenching pattern (Figure 4). As a result, value of *n* falls towards unity. The values of K_b_ and *n* at different temperatures are given in Table 3. The K_b_ values obtained by quenching method were found to be in the range of 0.26×10^4^–6.03×10^4^ M^−1^ which signifies moderate binding. K_b_ values for 1N and 2N increased on increasing the temperature (positive dependence) but for 8H it showed negative dependence. Thus the affinity of 1N and 2N to HSA increased with temperature and that of 8H decreased. As the temperature approaches to 45°C, a reversible separation of domain I and II takes place [21]. This domain separation probably induced relaxation in the binding constraints of 1N and 2N. The separation might as well create some more binding sites as is evident from the increasing values of *n* ranging from 0.83 to 1.21 (Table 3). Also, at higher temperatures a slight expansion of the binding site might provide a larger hydrophobic area for the lodging of more pollutant molecules. For 8H however decreasing affinity with increase in temperature suggests that domain separation may not be so favorable for its binding to HSA.

**Figure 6:**
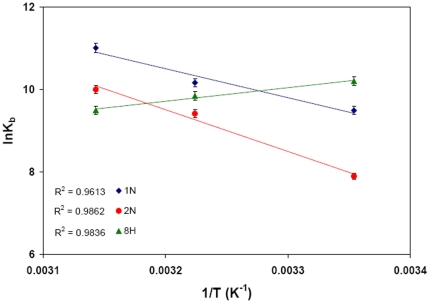
van't Hoff plot for temperature dependence of K_b_. Obtained from HSA fluorescence quenching by pollutants at 25, 37 and 45°C. [HSA = 2 µM; 1N = 2N = 8H = 0–50 µM].

Utilizing the binding constants at three temperatures, the thermodynamic parameters were determined from linear van't Hoff plot (Figure 6) and the observed values are presented in Table 3. The spontaneity of the HSA-pollutant interaction is represented by the negative values of ΔG. ΔH for HSA–1N and HSA–2N systems were found to be positive. Thus the formations of HSA–naphthol complexes were endothermic reactions accompanied by positive ΔS values. A positive ΔS value and a positive ΔH are frequently taken as a typical evidence for hydrophobic interaction [22]. Therefore, binding of naphthols to HSA may involve mainly non–polar hydrophobic residues of protein molecule. Meanwhile, it is also observed that the major contribution to ΔG arises from the ΔS rather than from ΔH that makes the binding process to be entropy driven. But in case of 8H, ΔS and ΔH values were negative with ΔH as major contribution to ΔG. This indicates that HSA–8H interaction is enthalpy driven and should interact with the protein through hydrogen bonds.

Even the values of thermodynamic properties of protein-ligand interactions may change if we use different formula based approaches in fluorescence spectroscopy. When we applied Equation 3 for the determination of ΔG from K_b_ values, we got similar values of ΔG as in absorption spectroscopy. Further, these values were plotted against temperature (Equation 8) to find out ΔH and ΔS from the intercepts and slopes respectively (Figure S1). If we use the same K_b_ values as above for van't Hoff Equation 7 to get ΔH and ΔS and ultimately the values of ΔG (Equation 8), these values are far different from the previous approach (Figure 6 and Table 3). Here we found the ambiguity even in the data obtained from fluorescence spectroscopy only.

### Energy transfer between HSA and pollutants

**Figure 7:**
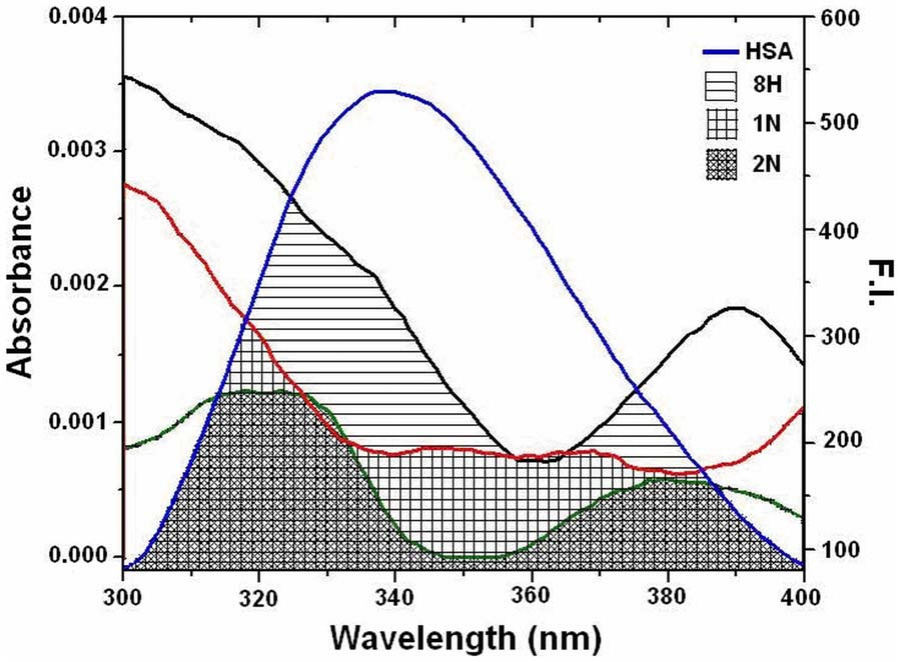
Tryptophan fluorescence resonance energy transfer. Spectral overlap of the fluorescence emission of HSA (λ_ex_ = 295 nm) and absorption spectra of pollutants [HSA = Pollutants = 2 µM].

A possibility of energy transfer between HSA and pollutants was investigated to further confirm the proximity of binding pollutants to the protein. Figure 7 shows the spectral overlap between the emission spectrum of HSA and the UV–absorption spectra of the pollutants (1N, 2N and 8H) with molar ratio of HSA: pollutant (donor: acceptor) as 1. As described in methods J, R_o,_ r and E values were derived from the overlapping spectral area and values for HSA–1N, HSA–2N and HSA–8H complexes are given in Table 4. R_o_ and r fall in the range of 3.28–4.00 nm and 4.39–5.77 nm respectively and these distances are just only the average values between bound ligands to the W214 of HSA and which were possibly affected by several factors when calculated by FRET theory. The energy transfer took place from HSA to pollutants with great possibility as in each case the distances between donor and acceptors were on the scale of 2–8 nm that satisfies 0.5R_o_<r<1.5R_o_ in accordance with Förster's non–radiative energy transfer theory [23], [24]. Also, range of r values do not exceed the dimensions of the protein (8×8×3 nm) [25] which shows that the energy–transfer from HSA to pollutants is possible when bound anywhere in the protein. This further justifies that the energy transfer between HSA and pollutants contributes to the noticeable decrease of protein fluorescence intensity through static quenching mechanism upon HSA–pollutant interactions. We however acknowledge the limitations with our energy transfer measurements where simultaneous anisotropy changes and the life time alterations in presence of pollutants could lead to a quenching or change in the quantum yield of the Tryptophan. These limitations complicate the measurement of the spectral overlap and that of measured distances between the pollutants and the protein. Therefore the distances measured here must be considered as an apparent measure of the protein-pollutant binding event.

### Identification of pollutant binding sites in HSA

**Figure 8:**
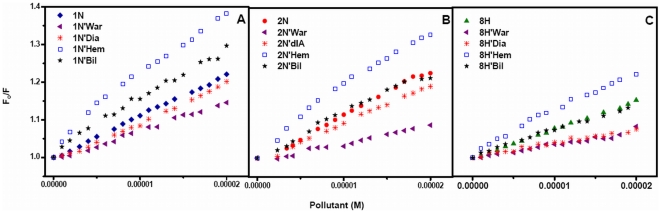
Competative binding of (A) 1N, (B) 2N and (C) 8H to HSA in the presence of site markers at 35°C. [HSA = 2 µM; site markers = 4 µM; 1N = 2N = 8H = 0–50 µM].

Even a minor modification in the structure and configuration of ligand can significantly affect the binding forces and may even lead to binding at an alternative site. This creates difficulty in the prediction of accurate binding site for a ligand. To facilitate the identification of the binding site, some probes are often used, which are already known to specifically bind to a known region on HSA and then their competition to these established ligands is investigated. It has been already established by X–ray crystallography studies that hemin is probe for subdomain IB [26], bilirubin is probe for the region between subdomain IB & IIA [27], warfarin is probe for subdomain IIA or Sudlow site 1 [28] and diazepam is probe for subdomain IIIA or Sudlow site 2 [29]. Experiments of pollutants binding competitively to HSA were performed by pre–saturating the protein molecule with hemin, bilirubin, warfarin and diazepam. In order to compare the effect of site markers on HSA–pollutant systems, the emitted fluorescence intensity data in the absence and presence of probes were plotted using Stern–Volmer Equation (Figure 8) and the results are summarized in Table 5. The decrease in fluorescence quenching by 1N, 2N and 8H in presence of warfarin and diazepam show that all these three pollutants compete with warfarin and diazepam. This suggests that these ligands bind to subdomain IIA in the Sudlow site 1 and to subdomain IIIA in Sudlow site 2, the respective binding sites of warfarin and diazepam.

However the presence of hemin and that of bilirubin show positive heterotropic cooperativity or allosteric activation for binding of 1N. This implies that albumin on interaction with these probes tends to have increased binding of 1N. This also demonstrates the non–occupancy of 1N to the subdomain IB and between subdomain IB & IIA, respective sites for hemin and bilirubin. The observed increase in binding of 1N further establish conformational alterations in albumin as binding of one ligand influences binding of the other. These allosteric effects were likely to result from conformational changes in protein with partially overlapping binding sites. Hence, the allosteric effects of hemin as well as bilirubin site on 1N binding site may also imply the sharing of a common face among these sites. This is in accordance with the work of Dröge *et al.* [30] where binding of site 2 marker increases the change in enthalpy for interaction of site 1 marker.

The tryptophan (W214) emissions of HSA–2N and HSA–8H systems in absence and presence of bilirubin were almost identical but in presence of hemin it resembled to HSA–1N system. Here only hemin induced conformational changes were effective in the enhancement of 2N and 8H binding to the HSA. Thus, ligands like hemin may prolong the storage period of the three pollutants in blood and facilitate in maximizing the effects of these pollutants on the organisms.

### Calorimetric investigation of HSA–pollutant association

**Figure 9:**
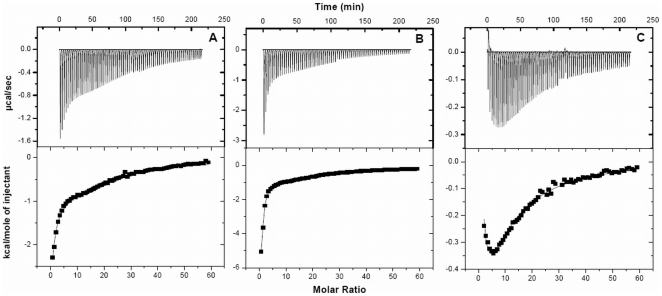
Isothermograms representing the binding of HSA to (A) 1N, (B) 2N and (C) 8H in 20 mM Tris–HCl pH 7.4 at 15°C. The upper panels represent the raw data and the bottom panels are the best fits of the raw data fitted to the multiple binding sites model. The concentration of protein was 24.4 µM and the pollutants were 3 mM. Appropriate background corrections were made to account for the heats of dilution and ionization.

**Figure 10:**
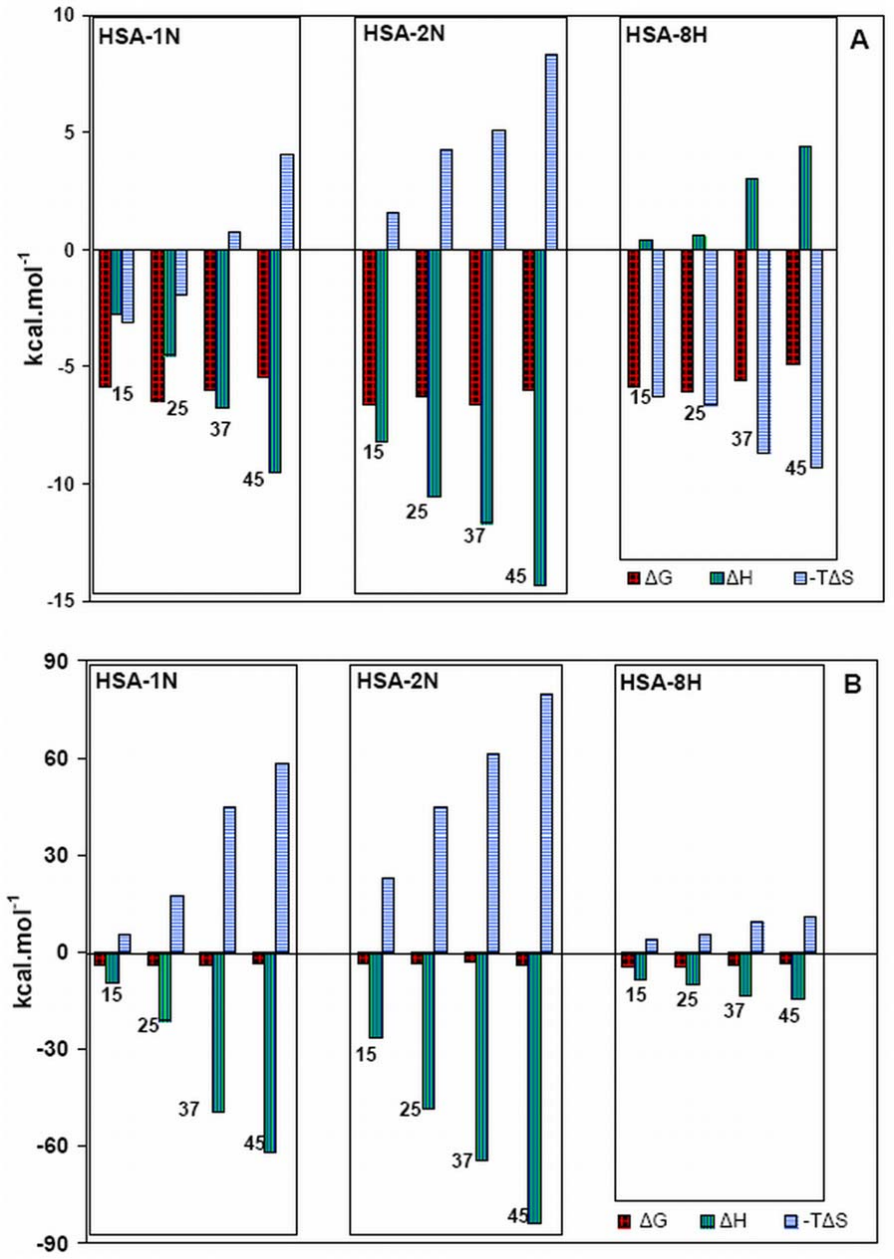
Thermodynamic signatures for 1N, 2N and 8H associations to HSA (A) for binding site 1 and (B) for binding site 2. The corresponding temperatures in °C are indicated.

Studies using isothermal titration calorimeter were carried out in order to further investigate the thermodynamics of HSA–pollutant interactions (Figure 9). Temperature dependence of protein–ligand interactions were monitored at 15, 25, 37 and 45°C. The results are summarized in Table 6. It is observed that the order of association constants (K_b_) for HSA–pollutant complex formation is 2N>1N>8H and 1N>8H>2N at site1 and site2 respectively. ΔG, ΔH and ΔS for complex formation are plotted as a function of temperature (Figure 10). The negative values of the interaction free energy change (ΔG) in each case of HSA–pollutant association reveal that binding occurs spontaneously. The negative ΔG value directly relates to the binding affinity and the stability of complex. Except for 1N and 8H binding at site 1, both the binding enthalpy and binding entropy were found to be negative. This is a usual observation in protein-ligand interaction [22] as a favorable binding enthalpy essentially results in greater entropic constraint leading to more unfavorable contribution to binding free energy. Though the binding enthalpies and entropies of 1N, 2N and 8H to HSA interaction at the two sites differ, their binding free energy changes (ΔG) remain effectively the same. This is because of enthalpy–entropy compensation (EEC) where changes in the binding enthalpy are well compensated by changes in the binding entropy. The protein–ligand interaction at site 2, (Figure 10 (B), Table 6), binding enthalpy and entropy show greater variation than the total free energy which implies significant EEC at site 2 [31].

Except for 8H binding at site 1, the negative enthalpy of reaction was observed to increase with increase in temperature that suggests that the interactions were enthalpy-driven. However at higher temperatures the HSA–pollutant complexation, except for binding of 8H at site 1, is shifted towards entropically favored association. It is possible that larger entropy of water at subdomain IIA and a greater hydrophobic character of 8H could be responsible for entropically-driven interaction of 8H at site 1. Also, binding of 1N at site 1 is observed to have a peculiar transition as a function of temperature (Figure 10A). Its association to HSA at ∼34°C (T_S_) is totally driven by enthalpy as entropy approaches zero. In all the other cases of HSA–pollutant interaction the T_S_ is below 15°C. Also, temperature at which the enthalpy contribution becomes negligible (T_H_) falls below 15°C in all cases of the present study.

**Figure 11:**
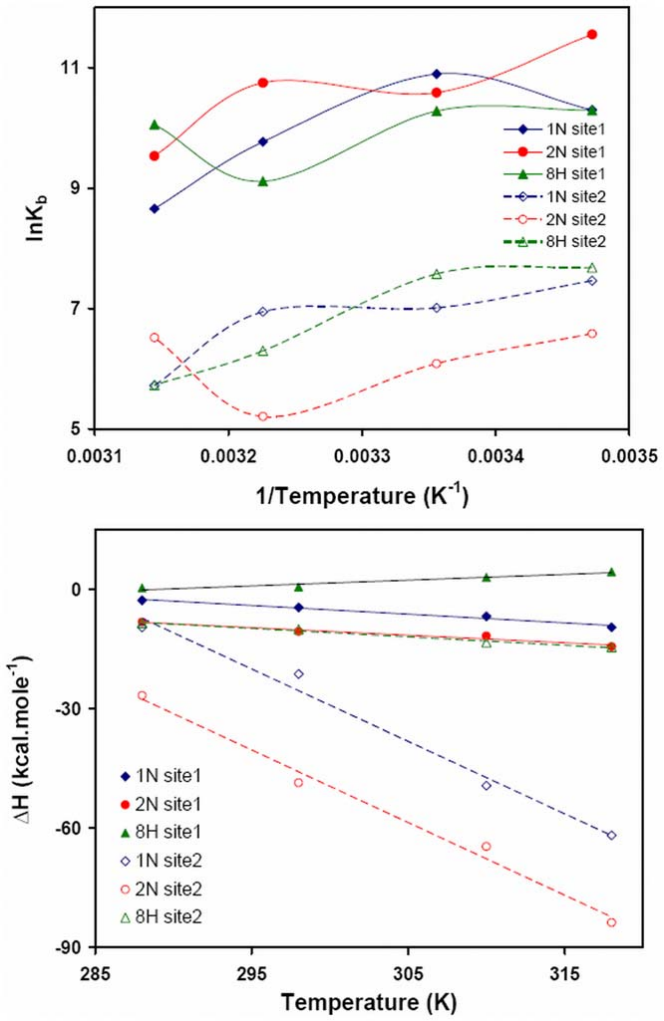
Temperature dependence of thermodynamic parameters obtained by ITC. (A) Temperature dependence of ΔH for the determination of 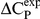; (B) van't Hoff plot for temperature dependence of K_b_.

The van't Hoff plot or the temperature dependence of pollutant binding constants to each site of HSA is shown in Figure 11A. The observed non–linearity of the van't Hoff plot may be due to linkage of other processes such as conformational changes occurring during protein–ligand interaction [32]. The first derivative of temperature dependence of enthalpy change is used for the calculation of experimental heat capacity change (ΔC_p_^exp^). From the slope of ΔH *vs* temperature (Figure 11B), the obtained ΔC_p_^exp^ for binding of 1N, 2N and 8H at site 1 were −0.2187, −0.1896 and 0.1432 kcal.mol^−1^.K^−1^ respectively whereas for site 2 these values were −1.819, −1.8227 and −0.2122 kcal.mol^−1^.K^−1^ respectively (Table 6). ΔC_p_^exp^ is comparatively larger and negative at site 2 that indicates a larger change in solvent exposed surface area in domain III. A negative ΔC_p_^exp^ also signifies specific binding of ligand accompanied by burial of non–polar surface [33], [34].

The thermodynamic signatures of protein–ligand interactions impersonate the type of forces responsible for ligand association [22]. In this process the uptake/release of water and ion molecules, the restriction of degrees of freedom of polypeptide main chain and side groups or minimal loss of conformational degrees of freedom, changes in vibrational content, burial of water–accessible surface area and hydrophobic interactions sum up to give net entropy contribution. The H–bond formation and van der Waals interactions sum up to provide enthalpy contribution to free energy of association. On the basis of these findings we have inferred the forces responsible for corresponding HSA–pollutant interactions and are described in Table 6.

### Molecular Docking

**Figure 12:**
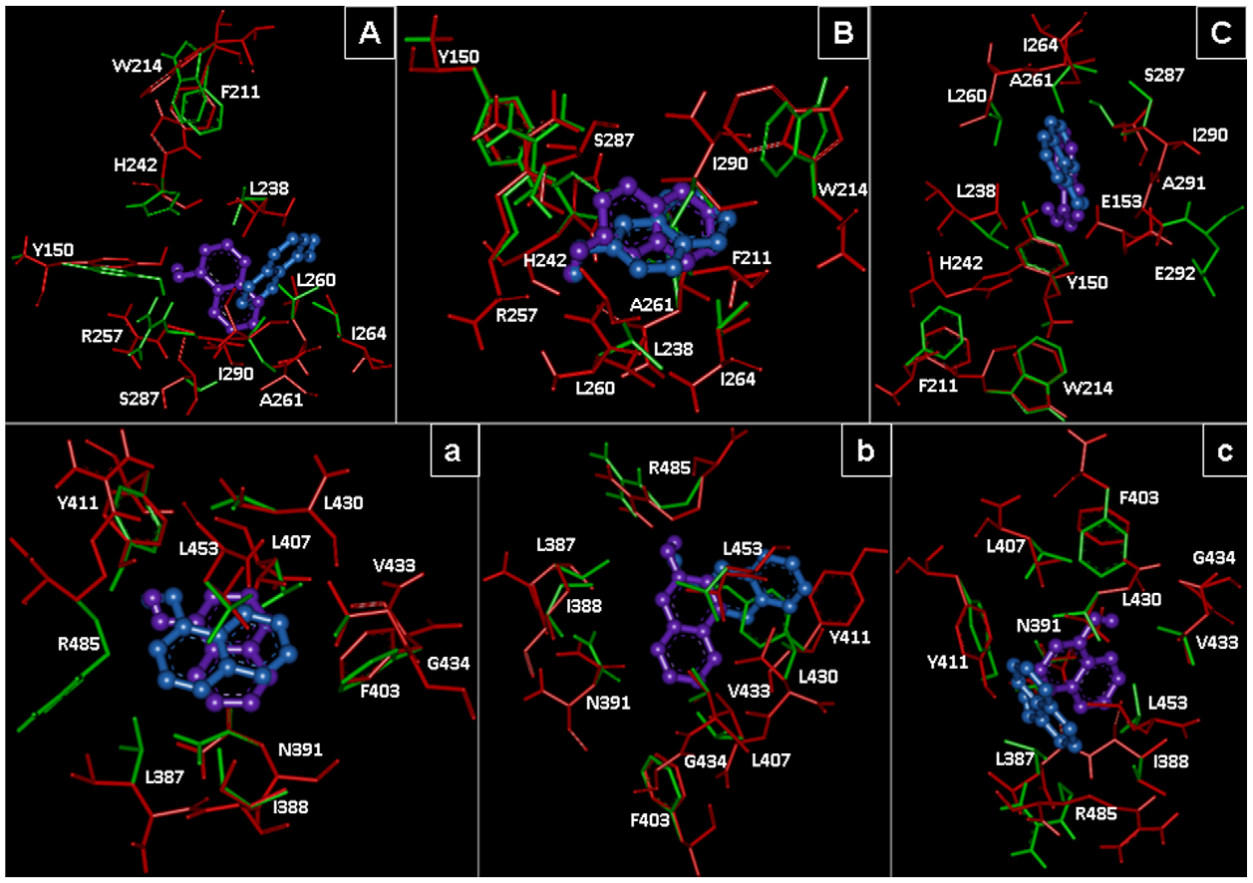
Molecular docking of pollutants to HSA molecule. Residues in flexible (red) and rigid (green) modes are shown in site 1: (A) 1N, (B) 2N, (C) 8H and site 2: (a) 1N, (b) 2N, (c) 8H. In flexible docking the ligands are in purple and in rigid docking they are in blue color.

**Figure 13:**
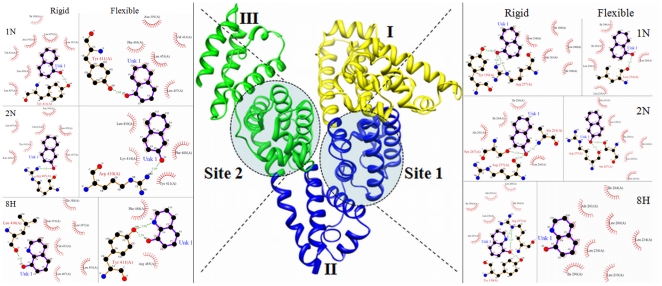
Molecular contacts between the amino acid residues of site 1 (right side) and site 2 (left side) of HSA and pollutants (1N, 2N and 8H) within 5 Å. Obtained from rigid and flexible docking by AutoDock4 are shown in the form of LigPlot.

For the better understanding of HSA–pollutant binding the complementary applications of molecular docking of pollutants on HSA has been performed with Autodock simulation analyses in both rigid and flexible conditions. As in most of the studied cases the docking simulations are based upon the protein crystal structure in rigid state. But upon ligand binding the proteins acquire different conformations. Unless significant freedom was allowed in the structural flexibility of the protein, it is unlikely these results yield useful information about the specific residues involved in the interactions with the pollutants if they are supposed to dock only flexible ligand and a rigid protein. Hence, both ligand and protein molecules were set to be in flexible mode. These two different conditions were considered. The best energy ranked results are shown in Figure 12 and Figure 13 and are summarized in Table S1 and Table S2. The flexible docking results suggest that both site 1 and site 2 could occupy all the three pollutants 1N, 2N and 8H but shared different binding region in the same site. The binding constants (K_b_) obtained from flexible molecular simulations (Table S1) reveal that the pollutants bind loosely at the peripheral side of the cavity at site 1 (K_b_ in the order of 10^5^) whereas they bind tightly and deep inside at site 2 (K_b_ in the order of 10^6^). At site 1, variations in the water structure may help to make the pocket lesser adaptable to ligands [29]. The interactions between the HSA and pollutants were exclusively hydrophobic in nature as reflected by several non–polar (F211, 223; W214; A215, 261, 291; L219, 238, 260; V241; I264, 290), one polar (S287) and few charged (R218, 222, 257; H242) residues at site 1. Similarly site 2 is made of several non–polar (F488; L387, 430, 453; I388; A449; V485, P384), one polar (Y411) and few charged (E450, 489; N391, R411, 485) residues. These residues were in the proximity distance of 5 Å of the bound ligands. Although the involvement of non polar residues makes the interactions to be hydrophobic in nature but the strong intermolecular H–bonding possibility between pollutants and HSA also exists. In flexible docking binding of 8H at site 1 doesn't involve any H–bond and is stabilized by hydrophobic interactions only (Table S1) whereas in case of rigid docking 8H forms H-bond with Y150 and R257 (Fig. 13 & Table S1).

Further identification of amino acid residues involved in ligand interaction, the accessible surface area (ASA) of protein before and after ligand binding were calculated (Table S1). It was found that in flexible docking the ΔASA of L238, 260, I264, 290, N391, Y411, V433, L453 for 1N; L238, 260, 430, R257, 410, I290, A291, Y411, F488 for 2N; and L219, 238, I290, Y411, R485, F488 for 8H were ranged from 10.53 to 41.52 Å^2^. Hence, these residues were observed to take part in pollutants binding to HSA through H–bonding and hydrophobic interactions. These ligands occupied a small space in the cavity as was evident by the observation that maximum four residues were strongly involved in the hydrophobic complexation (from ΔASA results) while there existed an involvement of eleven residues for site 1 marker warfarin, and seven residues for site 2 marker diazepam. This is the reason why warfarin (and up to some extent diazepam too) is able to effectively compete with the binding pollutants (Figure 8). The change in Gibbs free energy (ΔG) of HSA–pollutant interactions calculated from computational approach range from −7.2 to −8.3 kcal.mol^−1^ that was higher than the experimental values obtained from spectroscopy and ITC. ΔC_p_^calc^ values (from Equation 14) are very small and in the range of 0.010–0.039 kcal.mol^−1^K^−1^ (Table S1). Thus results obtained from docking also indicated that the HSA–pollutant interactions were dominated by hydrophobic forces.

### Conformational changes in HSA upon pollutant interactions

To determine whether pollutants affect the structure of HSA molecule or not, the qualitative features of UV–visible and fluorescence spectroscopy as well as thermodynamic signatures of HSA in absence and presence of pollutants were also analysed. From Figure 1 it is clear that the spectral intensity in far–UV region decrease with a red shift in wavelength maxima. As this is the absorption region of peptide bond, the change in secondary structures of protein can be deduced. Hence, the loops and α–helical contents of HSA in HSA-pollutant complex vary from that of free HSA. The changes in absorption intensity and the wavelength shifts are attributed to the change in H–bond rearrangements and change in polarity of microenvironment of aromatic residues [35] leading towards change in secondary as well as tertiary structures of HSA. Thus the results of absorption studies indicate that the solvation shell and the intramolecular or intermolecular association of the chromophoric groups on HSA may be partly or totally altered in the presence of these pollutants which lead to an altered solute–environment interaction.

The blue or red shift in wavelength maxima of fluorescence spectra is a better probe to determine the change in tertiary structure of the protein. The change in polarity in the vicinity of tryptophan due to change in the microenvironment of protein is main cause of the shift in wavelength maxima. A similar shift in wavelength maxima is clearly observed in presence of pollutants (Figure 3). Studies looking further into details of pollutant–induced conformational and functional alterations in HSA are currently undertaken by authors.

## Discussion

Considering the advantages of calorimetry, this is the first report of its kind that investigates comprehensively the thermodynamics of HSA–pollutant association. The results were further compared with spectroscopic observations in the identical conditions of complex formation. Furthermore, the obtained results from spectroscopy and calorimetry were validated from bioinformatics tools.

The considered pollutant molecules are hydrophobic molecules that are sparingly soluble in water. The hydrophobic core of the protein provides an excellent site for the uptake of these water–insoluble hydrophobic molecules from the aqueous bulk. This is similar to the partitioning of organic molecules into microscale admicelles [36]. Naphthols turn out to be of moderate binding with HSA as the association constants range from 10^4^ to 10^5^ M^−1^ as compared to those for strongly bound ligand–protein complexes that vary within the range of 10^6^ to 10^8^ M^−1^ [37]. This also signified that the HSA–pollutant complexes formed were weak in nature and could be readily displaced by other competing compounds having higher binding constants or if present at higher concentrations.

**Figure 14:**
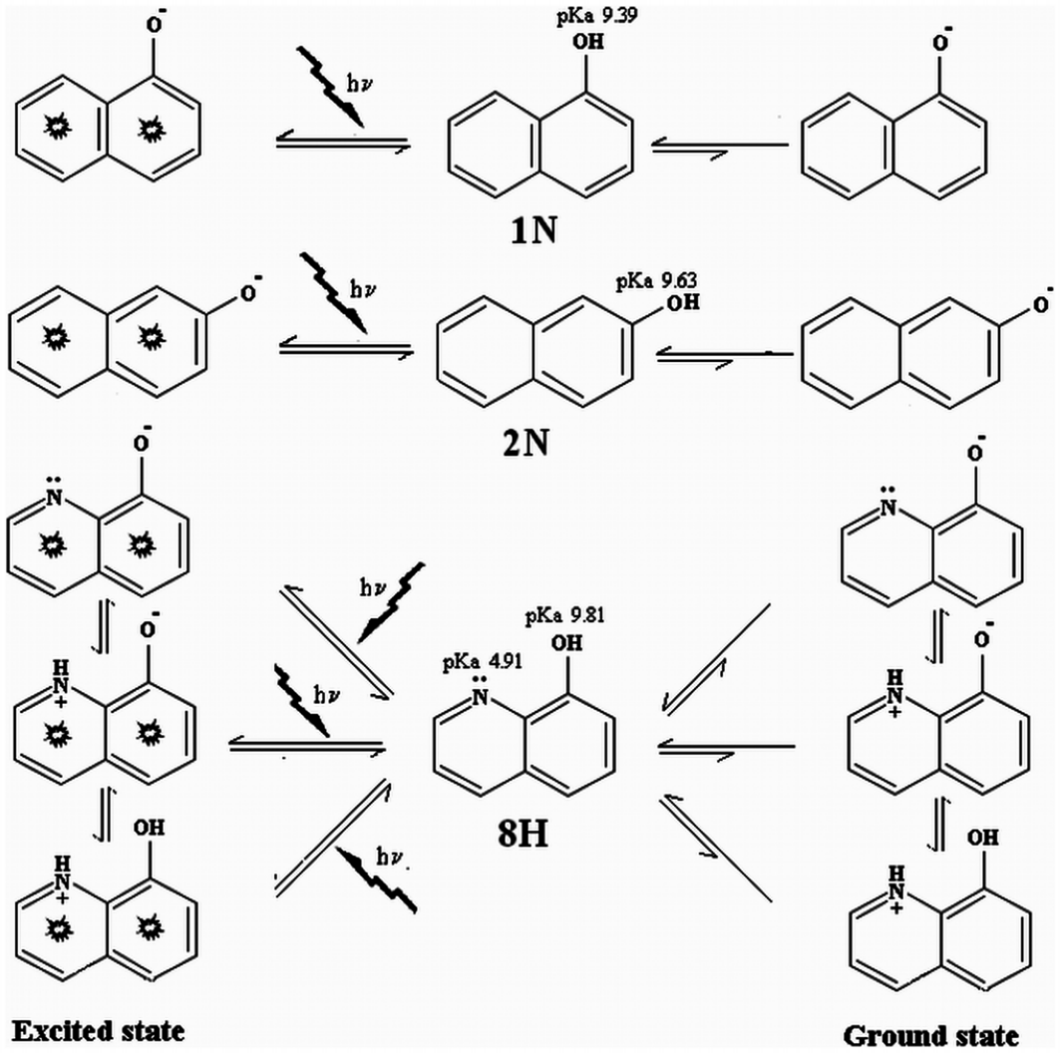
pKa dependent dissociation equilibrium of pollutants in ground and excited states. In ground states the equilibrium shifts predominantly towards non-ionic species whereas in excited states the equilibrium is maintained between non-ionic and ionic species.

HSA comprises of three homologous domains: domain I (residues 1–195), domain II (196–383) and domain III (384–585). Each domain is a product of two subdomains A and B that comprises common structural motifs with six and four α–helices in subdomain A and B respectively. HSA has two different Sudlow's binding pockets namely binding site 1 and binding site 2 that bind to a variety of ligands. The entrance of site 1 is surrounded by positively charged residues R257, R222, K199, H242, R218 and K195, and the inside wall of the pocket is formed by hydrophobic side chains of Y150, F211, F223, W214 etc. Hence, anionic and neutral ligands show a significant interaction with binding site 1. Since there exists equilibrium between non–dissociated and dissociated forms of 1N, 2N and 8H (Figure 14), therefore both anionic and neutral form of all three pollutants can interact with site 1. Site 1 is larger, flexible and more adaptable. There is somehow compassion in stereoselectivity of ligands interacting with this site. This is evidenced by the two enantiomers of warfarin (R&S) binding in almost similar manner. This may be the reason that 1N and 2N as well as 8H binds perfectly in this site without showing much difference in their affinity. At site 2 R410 is located at the mouth and the pocket is lined by hydrophobic side chains facing the –OH of Y411 and of S489, –COO^−^ of E450. Therefore positively charged ligands having H–bonding donor or acceptor atom can preferably bind in site 2. Only 8H among the three considered pollutants exists as positively charged molecule at physiological pH (Figure 14), therefore, it was maximally inhibited (45%) by a positively charged site 2 probe diazepam (Figure 8, Table 5). Site 2 is smaller, lesser flexible, more restricted and ligand binding here seems to be governed strictly by stereoselectivity. This is evidenced by the observation that affinity of L–Trp is 100 times greater than the D–Trp. This is further confirmed with our results where among 1N and 2N, only 1N is able to perfectly bind at site 2. K_b_ for 2N here is estimated to be 10^2^ which is the minimum affinity that can be estimated by the current ITC instrument. Therefore, these low binding values can be assumed to be non-specific and insignificant (as shown also by molecular docking methods).

As noted earlier, among the pollutants 1N and 2N are configurational enantiomers with all the three pollutants being isoelectronic. However the distribution of electrons around these molecules is different. The distribution of electrons may undergo a rearrangement in the presence of light due to an alteration in proton dissociation properties of these pollutants in aqueous medium where these pollutants are in equilibrium with respective conjugate base. As the physiological pH (7.4) is lower than the pKa of pollutant –OH group (Table 1), the protonated forms will predominate. Also these pollutants when excited during spectrophotometry have larger dissociation constants. For example, the dissociation constant of 2-naphthol is larger in the excited state i.e. it is a stronger acid in its excited state. The literature values for the ground and excited state acid equilibrium constants are 3.1×10^−10^ and 2.0×10^−3^ [38]. This results in lowering of pKa leading to a predominance of unprotonated form (Figure 14). Hence, the dissociation depends on whether these pollutants are in ground state or in excited state. In spectroscopic studies of HSA-pollutant complexation the pollutant molecules may also attain in their excited state leading to an accumulation of unprotonated ionic species. This excitation and the subsequent shift in ionic population of pollutants don't occur during calorimetric studies. Thus, in spectroscopic and calorimetric observations the compounds were in excited and in ground state respectively. We suggest that this may be the reason for differences observed in binding isotherms and values of thermodynamic parameters obtained by spectroscopy and calorimetry. Moreover, the dielectric constants of these pollutants are quite low (Table 1) in comparison to water (∼80). As the obtained data from UV–visible spectroscopy show dielectric dependency of medium in which the experiment was performed, the observed wavelength shift in UV–absorption spectra of HSA–pollutant complex HSA can also be due to a change in dielectric constant of the microenvironment of the protein suspended in aqueous medium. Dielectric dependency of UV absorption may also affect the extent of fluorescence emission that is used to calculate the binding isotherms of protein–ligand association. Furthermore, in spectroscopic approaches protein (P)–ligand (L) equilibrium binding assay directly yield values for [PL], [P], or [L] as a function of the signal change and its degree of saturation. Subsequently, K_b_, ΔH, ΔS as well as ΔG at different temperature were calculated using Equations 3, 6, 7 and 8. Such a non–calorimetric approach to the thermodynamics [39] has ruthless shortcomings where K_b_ usually appears temperature–independent. This happens as the enthalpy-entropy compensation and experimental errors propagate into large miscalculation of temperature–dependent ΔH and ΔS. Therefore, non–calorimetric determination of binding energetics is frequently blemished.

**Table 1:** Physicochemical properties of pollutants.

Properties	1–naphthol (1N)	2–naphthol (2N)	8–quinolinol (8H)
Empirical formula	C_10_H_8_O	C_10_H_8_O	C_9_H_7_NO
IUPAC name	naphthalen–1–ol	naphthalen–2–ol	quinolin–8–ol
M.W.	144.16	144.16	145.16
LogP	2.84	2.7	1.7
pKa	9.39	9.63	9.81 and 4.91
Polar surface area	20.2	20.2	33.1
H-Bond Donor	1	1	1
H-Bond Acceptor	1	1	2
Complexity	133	133	138
Dielectric constant	5.03	4.95	—

**Table 2:** Binding parameters of naphthol titrations to HSA in 20 mM Tris–HCl pH 7.4 at 37°C from UV–visible spectroscopic measurements.

Pollutants	P∶L for saturation*	*h*	K_b_ (×10^4^ M^−1^)	ΔG (kcal.mol^−1^)
**1N**	1∶8	0.99	1.03	−5.67
**2N**	1∶11	1.00	1.49	−5.89
**8H**	1∶40	0.99	0.19	−4.66

*The saturation ratios were calculated from the obtained values of ΔA∞.

**Table 3:** Binding parameters of pollutant interaction to HSA in 20 mM Tris buffer pH 7.4 at different temperature obtained and calculated from fluorescence quenching results.

Ligand	T(°C)	*n*	K_sv_(×10^4^ M^−1^)	K_q_(×10^12^ M^−1^ S^−1^)	K_b_(×10^4^ M^−1^)	ΔG*(kcal.mol^−1^)	ΔH*(kcal.mol^−1^)	ΔS*(cal.mol^−1^.K^−1^)	Dominating forces involved(inferred)
**1N**	25	1.003	1.27	2.96	1.32	−5.61 (−1.40)	21.49 (3.52)	89.47 (16.57)	Hydrophobic interactions
	37	1.078	1.15	2.67	2.59	−6.25 (−1.60)			
	45	1.218	0.92	2.14	6.03	−6.95 (−1.74)			
**2N**	25	0.835	1.46	3.39	0.26	−4.65 (−1.18)	20.30 (5.09)	83.89 (21.09)	Hydrophobic interactions
	37	1.007	1.13	2.62	1.22	−5.79 (−1.44)			
	45	1.082	0.95	2.20	2.19	−6.31 (−1.61)			
**8H**	25	1.102	1.16	2.69	2.68	−6.03 (−1.54)	−6.65 (−1.65)	−2.06 (−0.40)	H-bonding, conformational change
	37	1.047	0.85	1.98	1.75	−6.01 (−1.53)			
	45	1.010	0.70	1.64	1.32	−5.99 (−1.52)			

*ΔH & ΔS in parentheses are derived from van't Hoff (Equation 7) and ΔG in bracket is derived from (Equation 8). The unit of Temp., K_sv_, K_q_, K_b_, ΔG, ΔH and ΔS are °C, M^−1^, M^−1^ s^−1^, M^−1^, kcal.mol^−1^, kcal.mol^−1^ and cal.mol^−1^.K^−1^ respectively.

**Table 4:** FRET data obtained from spectral overlap of HSA emission and pollutant absorption.

Variables	1N	2N	8H
**E**	0.109081	0.148485	0.100669
**J** (cm^3^ M^−1^)	11.20×10^−14^	5.77×10^−14^	19.03×10^−14^
**R_o_** (nm)	3.66	3.28	4.00
**r** (nm)	5.20	4.39	5.37

**Table 5:** The competitive experiments of HSA–pollutant system with different site markers.

Category	Site Markers	Probes for	Binding Constants(M^−1^)$	Inhibition of Pollutant binding by these probes*	Enhancement of Pollutant binding by these probes#
Non-drugs	Hemin	Subdomain I (IB)	1.1×10^8^	1N (0%)	1N (80%)
				2N (0%)	2N (40%)
				8H (0%)	8H (50%)
	Bilirubin	b/w Subdomain IB & IIA	9.5×10^7^	1N (0%)	1N (35%)
				2N (0%)	2N (0%)
				8H (0%)	8H (0%)
Drugs	Warfarin	Subdomain IIA (Sudlow site 1)	3.3×10^5^	1N (30%)	1N (0%)
				2N (70%)	2N (0%)
				8H (50%)	8H (0%)
	Diazepam	Subdomain IIIA (Sudlow site 2)	3.8×10^5^	1N (10%)	1N (0%)
				2N (10%)	2N (0%)
				8H (45%)	8H (0%)

$ Binding constants of these probes to HSA as taken from other reported studies; hemin [46], bilirubin [47], warfarin [48] and diazepam [49].

*1N/2N/8H mediated W214 fluorescence quenching was *inhibited* by these site markers up to this %.

# 1N/2N/8H mediated W214 fluorescence quenching was *enhanced* by these site markers up to this %.

Looking further in to the differences in results obtained from calorimetric, spectroscopic and computational methods, we observed that the thermodynamic signatures obtained from ITC experiments suggest that HSA–1N interaction involves H–bonding as well as the conformational changes (Table 6) where as the molecular docking simulation reveals no such H–bond. In a similar way, the binding isotherms obtained from fluorescence quenching data suggested that only hydrophobic interactions were responsible for interaction of 1N and 2N to HSA which again conflicted the calorimetric results and indicated that H–bonding was possible. However the formation of H–bonds between HSA and pollutants can be explained on the basis of water molecules. In HSA matrix, the trapped water molecule found near W214 drives the dipole–dipole interaction with W214 in subdomain IIA [40] giving it a peculiar conformation. Therefore, change in dielectric medium upon interaction of foreign ligands revealed that the reorientation of trapped water molecule would be allowed. Also, Crystallographic structure of HSA (PDB: 1E78) shows that K199 is arranged near this trapped water molecule [41]. K199 would give the determinant effect on the local conformation near water through its amino groups. As shown in Table S1 there is an involvement of K199 in 2N interaction and upon 2N interaction the water molecule can move and reorient to rearrange the H–bonding patterns of water to protein molecule that will lead to change in dipole–dipole relaxation and a conformational change subsequently. The –OH group of 2N may also be indirectly involved in energy and motional relaxation of water molecule as well as the dielectric relaxation in vicinity of water molecule. Here, the specific H–bonding patterns between HSA–pollutants determine the orientation of pollutants in the binding sites where each pollutant molecule adopts a different orientation in order to permit an H–bond with polar and charged residues, hence, R257 in site 1 and R410 and Y411 in site 2 may determine the ligand orientation. Thus, despite the contradicting results obtained from different experimental approaches, involvement of H–bonds between side chain of amino acid residues and ‘O’ and ‘N’ atoms of pollutants and therefore the electrostatic forces in HSA–pollutant complexation should not be ignored.

**Table 6:** Thermodynamic parameters of pollutants binding to HSA in 20 mM Tris-HCl pH 7.4 obtained and calculated by ITC data.

Site	Pollutant	Temp.(°C)	K_b_(M^−1^)	ΔH(kcal.mol^−1^)	TΔS(kcal.mol^−1^)	ΔG(kcal.mol^−1^)	ΔCp(kcal.mol^−1^.K^−1^)	Dominating forces involved(inferred)
1	1N	15	2.97×10^4^±2.2×10^3^	−2.76±0.10	3.13	−5.89	−0.2187	H-bonding, hydrophobic interactions
		25	5.38×10^4^±3.9×10^2^	−4.52±0.08	1.93	−6.45		H-bonding, hydrophobic interactions
		37	1.75×10^4^±1.1×10^3^	−6.77±0.08	−0.76	−6.01		H-bonding, conformational change
		45	5.76×10^3^±1.5×10^2^	−9.52±0.17	−4.05	−5.47		H-bonding, conformational change
	2N	15	1.04×10^5^±2.2×10^3^	−8.19±0.06	−1.57	−6.61	−0.1896	H-bonding, conformational change
		25	3.96×10^4^±1.9×10^3^	−10.56±0.22	−4.29	−6.26		H-bonding, conformational change
		37	4.66×10^4^±7.6×10^2^	−11.69±0.06	−5.05	−6.63		H-bonding, conformational change
		45	1.38×10^4^±4.5×10^2^	−14.36±0.27	−8.33	−6.02		H-bonding, conformational change
	8H	15	2.95×10^4^±7.2×10^2^	0.38±0.05	6.26	−5.88	0.1432	Hydrophobic interactions
		25	2.91×10^4^±4.4×10^2^	0.57±1.08	6.65	−6.08		Hydrophobic interactions
		37	9.07×10^3^±2.2×10^2^	3.05±1.49	8.66	−5.61		Hydrophobic interactions
		45	2.33×10^4^±2.2×10^3^	4.42±0.10	9.31	−4.89		Hydrophobic interactions
2	1N	15	1.74×10^3^±190	−9.54±1.03	−5.28	−4.26	−1.819	H-bonding, conformational change
		25	1.11×10^3^±57	−21.26±1.22	−17.11	−4.15		H-bonding, conformational change
		37	1.04×10^3^±40	−49.3±0.38	−45.03	−4.27		H-bonding, conformational change
		45	3.06×10^2^±23	−61.77±0.51	−58.16	−3.61		H-bonding, conformational change
	2N	15	7.24×10^2^±62	−26.62±0.82	−22.86	−3.76	−1.8227	H-bonding, conformational change
		25	4.40×10^2^±51	−48.54±1.64	−44.94	−3.6		H-bonding, conformational change
		37	1.83×10^2^±5.8	−64.58±0.84	−61.38	−3.2		H-bonding, conformational change
		45	6.77×10^2^±11	−83.66±4.80	−79.55	−4.11		H-bonding, conformational change
	8H	15	2.17×10^3^±110	8.47±0.14	−4.08	−4.39	−0.2122	H-bonding, conformational change
		25	1.95×10^3^±140	−10.04±1.00	−5.56	−4.48		H-bonding, conformational change
		37	5.45×10^2^±30	−13.33±1.44	−9.45	−3.88		H-bonding, conformational change
		45	3.08×10^2^±22	−14.54±0.23	−10.92	−3.62		H-bonding, conformational change

The internal dynamics of protein is affected by interaction of ligands [42] which affects the energetics of protein–ligand association because the accessibility to solvent of binding cavities is a subject of issue in which rapid exchange with pollutants necessitates a noteworthy role for conformational dynamics in order to access the pollutant molecules [43]. Hence, the decrease in configurational freedom of protein molecule upon complexation, ultimately, the loss of side chain and backbone entropy upon binding is assigned by positive value of ΔC_p_, and this positive value is an indication of the exposure of protein hydrophobic surfaces [44]. Whereas, a relatively large but negative contribution to the ΔC_p_ arises from the desolvation of exposed nonpolar groups upon ligand binding. From ITC the positive ΔC_p_^exp^ is shown only by HSA–8H in site 1 (smallest in magnitude) and other were in negative while from molecular docking simulations all pairs show negative ΔC_p_^calc^ except HSA–2N in site 1. The estimated values of ΔC_p_^calc^ based on the values of ΔASA_non–polar_ and ΔASA_polar_ obtained from molecular docking were different in signs and far smaller in magnitude than the ΔC_p_^exp^ obtained from ITC. This difference may be caused by binding induced flexibility change and protonation/deprotonation effect of proteins and pollutant molecules. UV–visible spectra and thermodynamic signatures reveal about the pollutant–induced conformational changes in HSA. In addition, at physiological pH although the pollutant molecules were predominantly in neutral form but equilibrium between protonated and deprotonated species exists (Figure 14) whereas in docking studies the molecules were taken only in their neutral form.

EEC is a common phenomenon especially where water molecule involves in binding where the enthalpic gain from a H–bond by itself is counterbalanced by the dehydration penalty for burying polar chemical functions and entropic losses from burial of the involved groups. In addition, EEC is ubiquitous in the association reactions which occur through non–covalent interactions [45]. This EEC effect is often endorsed to an exchange in which a larger enthalpic interaction results in a compensating loss of entropy due to motional constraints. In this study, the observed difference in EEC effects seems to be due to difference in the solvation energies of the pollutants.

From energy transfer studies, higher value of E and the smaller value of r for HSA–2N system as compared to HSA-1N and HSA-8H system were indication of a closer association and possibly higher perturbation of the structure. Hence, for 1N and 8H, thermodynamics and molecular docking approaches advocate for sequential binding in two sites of HSA but UV–visible absorption and fluorescence quenching (spectroscopy) insist it to be in single site. The difference in the obtained binding site stoichiometry among experimental techniques (2 from ITC and 1 from spectroscopy) can be explained in another way. Spectrometrically, 1∶1 stoichiometry was determined, which could come from the possibility that the observed conformational changes result from only one of the binding sites. With fluorescence the observation of only one site could come from a photo physical problem, because only one tryptophan is available for quenching or energy transfer. Upon the first binding event, the W214 is quenched to completeness, or in the case of FRET, the W214 can donate to only one of the acceptors at one time; thus, the second binding event becomes invisible.

The lower affinity of pollutants to HSA at higher temperatures suggests that the temperature dependant binding of HSA–pollutant may be associated with the modulation of physiological to non–physiological changes and native to non–native protein structural changes exaggerated by pollutants.

Conclusively, HSA binding sites are used as a model for the development of rational drug design because of their ligand–binding specificity. These sites are flexible and do not response straightforward to ligand to occupy in the cavity. The difference in occupancy and difference in binding patterns of the same pollutants in two different sites are a matter of structural rigidity, packing interactions and a subject to movement in the dimensions of the two cavities where one site may undergo some more collapse or be more conformationally mobile than the other one due to the conformational heterogeneity of the two sites. The polar as well as hydrophobic nature of both HSA binding sites and pollutants provide a template that is similar to naturally occurring catalytic sites and their corresponding substrates where even a small variation in ΔG leads to discrepancies in estimation of the concerned forces. Hence, only thermodynamic and spectroscopic data are not enough for such calculations but in addition to these obtained calorimetric and non–calorimetric parameters the crystal structures of HSA–pollutant will grant a precise scaffold for the estimation of exact forces involved. Now such a complete study will improve the bioinformatics for predictions concerning probable conformational heterogeneities for different protein–ligand associations. Thus, our study may represent the molecular basis for the important function of the targeting and transport of pollutants as well as other ligands by serum albumin throughout the circulatory system. Besides, though there are great conformational and morphological differences in all protein–ligand pairs, the present study will be helpful in all protein–ligand studies as the comparative use of simplified approaches deal out the dissection of the molecular mechanisms of intermolecular association. Based on the results of the present study we have seen that not only a functional group replacement but translocation of the group in a ligand greatly affects the interaction with protein where HSA interacts to configurational enantiomers and isoelectronics in a different way. Here, even the results of identical study obtained from different techniques such as spectroscopy, calorimetry and bioinformatics were contradictory due to several factors and limitations of the used techniques. Therefore, although there are thousand explanations for an obtained result, whether that result is right or wrong, interdisciplinary approaches with great preventative measures are key requisite for the solution of a problem productively.

## Supporting Information

Material S1 Isothermal titration Calorimetry.
(PDF)

Figure S1 **Temperature dependence of ΔG (** **Equation 8** **).** Here ΔG values were obtained from Equation 3 of HSA fluorescence quenching by pollutants at 25, 37 and 45°C [HSA = 2 µM; 1N = 2N = 8H = 0–50 µM].
(TIF)

Table S1 Interaction profile of pollutants with HSA after ***flexible*** docking with Autodock where number of contacts for corresponding residues to the ligand are given in parentheses and residues corresponding to ΔASA>10 Å^2^ are in bold.
(PDF)

Table S2 Interaction profile of pollutants with HSA after ***rigid*** docking with Autodock where number of contacts for corresponding residues to the ligand are given in parentheses and residues corresponding to ΔASA>10 Å^2^ are in bold.
(PDF)
