# Supplementary material for: Stereo-Selectivity of Human Serum Albumin to Enantiomeric and Isoelectronic Pollutants Dissected by Spectroscopy, Calorimetry and Bioinformatics
Source: PLoS One. 2011 Nov 2;6(11):e26186. doi: 10.1371/journal.pone.0026186 (PMC3206814; doi:10.1371/journal.pone.0026186)
Supplement: Material S1 — Isothermal titration Calorimetry. (PDF) [file pone.0026186.s001.pdf]

SUPPORTING INFORMATION

Supplementary Material S1

Isothermal titration Calorimetry: For sequential binding, the binding constants are defined relative to the progress of saturation, such as

$$K_1 = [HP]/[H][P]; K_2 = [HP_2]/[HP][P] \quad \text{(eq. 1S)}$$

where P denotes pollutant molecule and H denotes the HSA molecule [1]. The heat content Q after any i^{th} injection is then expressed as:

$$Q = P_tV_o[F_1\Delta H_1 + F_2(\Delta H_1 + \Delta H_2)] \quad \text{(eq. 2S)}$$

where F_i is the fraction of total macromolecule having i bound ligands and V_o is the active cell volume, whereas ΔH_1 and ΔH_2 correspond to the enthalpy change associated with the binding of the first, and second pollutant molecule to the HSA. The pertinent calculated heat effect (ΔQ) for the ith injection is:

$$\Delta Q = Q(i) + dV_i/V_o\ [(Q(i) + Q(i-1))/2]-Q(i-1) \quad \text{(eq. 3S)}$$

which is then used in the Marquardt minimization algorithm to obtain best fitting values until constant $\chi 2$ values were achieved.

ITC is the only technique facilitating us to investigate directly the fundamental macromolecular physical forces in adequate detail by measuring the qualitative and quantitative thermodynamic parameters as binding free energy (ΔG), enthalpy (ΔH), and entropy (ΔS) since these parameters offer a full description of the energetics governing molecular interactions. Gaining a detail of protein-ligand interactions is quite momentous in view of the fact that the binding practice endow with the essential knowledge for the improvement of molecular design strategies in basic as well as applied science. To determine

the number of pollutant binding sites on the HSA the goodness of fit was determined. In each case of HSA–pollutant complex formation the data fit in single, double and sequential binding modes were analyzed to get the lowest value of χ^2 and found that two sequential binding sites were most appropriate.

It is well known that ligands induce larger conformational changes in proteins; hence, it is very difficult to differentiate whether the obtained thermodynamic parameters are associated only with binding reaction or with synchronous conformational changes. The inferred forces deduced from the thermodynamic signatures as well as entropy-enthalpy compensation (EEC) are some how helpful to sort out this sticky situation. Hence, for the accurate determination of the thermodynamics of protein-ligand interaction incorporated with ligand-induced conformational alterations, different contributions in the change of free energy, enthalpy and entropy must be evaluated before going in the detail at molecular and atomic level. The total ΔG obtained experimentally is sum of different contributions such as ΔG^{elec} includes the electrostatic interactions, ΔG^{cav} refers to the cavitation free energy on binding, ΔG^{conf} is the loss of side chain conformational entropy on binding (TΔS), ΔG^{rt} represents the loss of translational and rotational entropy on complex formation. The presence of structured water molecules taking part in the binding interface will contribute to the amount and type of surface area buried whether they were polar or non-polar for the calculation of solvent-accessible surface area.

The change in enthalpy of binding imitates about the loss of protein–solvent H- bonds, van der Waals (vdW) interactions, and formation of protein–ligand bonds, salt bridges and vdW contacts, as well as solvent reorganization near protein surfaces. The binding heat obtained from the experiment (ΔH^{exp}) is the combination of intrinsic enthalpy (ΔH^{int}); enthalpy change

from conformational changes (ΔH^{conf}) and binding enthalpy due to protonation effects (ΔH^{prot}).

The total entropic contribution (ΔS^{tot}) in a protein-ligand reaction is composed of ΔS^{solv} which arises from solvent release upon ligand binding due to burial of apolar surface area. ΔS^{conf} contributes from the structural rearrangement of protein and ligand induced by complex formation. ΔS^{rt} brings about the loss of translational and rotational degrees of freedom.

1. Sharma R, Kishore N (2008) Isothermal titration calorimetric and spectroscopic studies on (alcohol + salt) induced partially folded state of a-lactalbumin and its binding with 8-anilino-1-naphthalenesulfonic acid. J Chem Thermodyn 2: 1141–1151.
