# Supplementary figures and images for: Stereo-Selectivity of Human Serum Albumin to Enantiomeric and Isoelectronic Pollutants Dissected by Spectroscopy, Calorimetry and Bioinformatics
Source: PLoS One. 2011 Nov 2;6(11):e26186. doi: 10.1371/journal.pone.0026186 (PMC3206814; doi:10.1371/journal.pone.0026186)

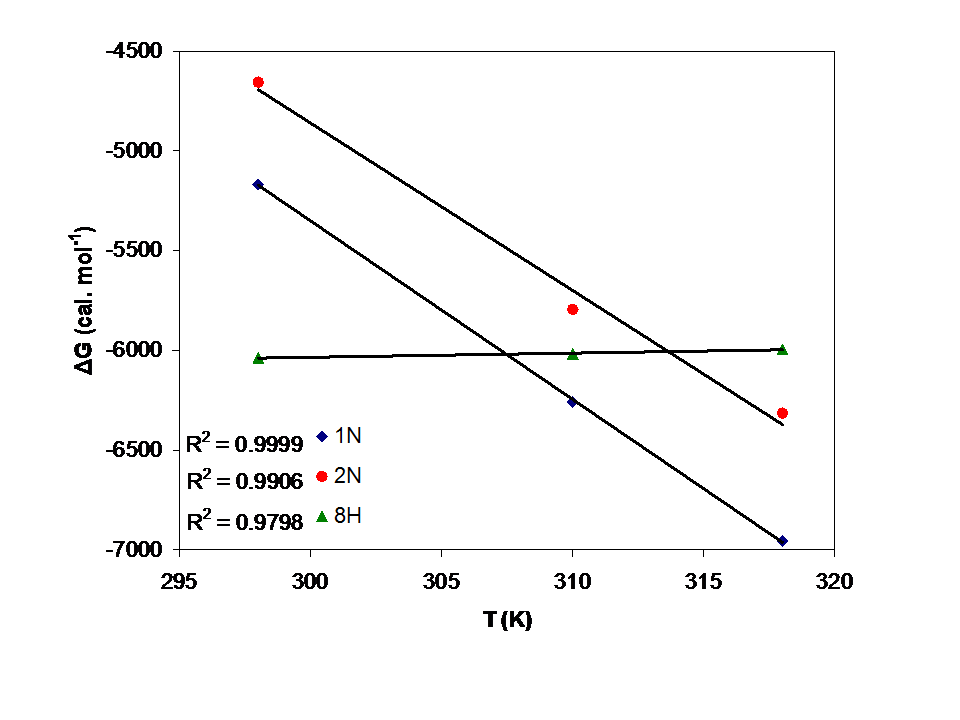

Supplement: Figure S1 — Temperature dependence of ΔG ( Equation 8 ). Here ΔG values were obtained from Equation 3 of HSA fluorescence quenching by pollutants at 25, 37 and 45°C [HSA = 2 µM; 1N = 2N = 8H = 0–50 µM]. (TIF) [file pone.0026186.s002.tif]
