# Supplementary material for: Stereo-Selectivity of Human Serum Albumin to Enantiomeric and Isoelectronic Pollutants Dissected by Spectroscopy, Calorimetry and Bioinformatics
Source: PLoS One. 2011 Nov 2;6(11):e26186. doi: 10.1371/journal.pone.0026186 (PMC3206814; doi:10.1371/journal.pone.0026186)
Supplement: Table S1 — Interaction profile of pollutants with HSA after flexible docking with Autodock where number of contacts for corresponding residues to the ligand are given in parentheses and residues corresponding to ΔASA>10 Å2 are in bold. (PDF) [file pone.0026186.s003.pdf]

Table S1. Interaction profile of pollutants with HSA after ***flexible*** docking with AutoDock where number of contacts for corresponding residues to the ligand are given in parentheses and residues corresponding to ΔASA>10 $Å^2$ are in bold.

Site	Pollutants	H-Bonding			Hydrophobic interactions			ΔG	K_b	ΔC_P^{calc}
		Residues	Average distance(Å)	ΔASA ($Å^2$)	Residues	Average distance(Å)	ΔASA ($Å^2$)	(kcal. mol^{-1})	(M^{-1})	(kcal.$mol^{-1}K^{-1}$)
1	1N	L234 (1)	3.13	2.77	L219 (3)	3.37	9.91	-7.23	2.00×10^5	0.038
					L234 (1)	3.46	2.77			
					**L238** (3)	3.70	26.83			
					**L260** (3)	3.55	10.65			
					**I264** (6)	3.72	16.29			
					**I290** (3)	3.50	18.48			
	2N	**R257** (1)	2.75	14.65	K199 (1)	3.81	2.11	-7.28	2.18×10^5	0.024
		S287 (1)	2.87	1.12	**L238** (2),	3.79	27.93			
					**L260** (1),	3.44	13.62			
					A261 (3),	3.60	3.69			
					I264 (2),	3.56	5.56			
					**I290** (7)	3.68	21.08			
					**A291** (3)	3.85	18.18			
	8H	-----	-----	-----	**L219** (2)	3.63	10.76	-7.96	6.84×10^5	0.039
					L234 (3)	3.77	4.38			
					**L238** (2)	3.84	29.78			
					L260 (6)	3.65	9.56			
					A261(2)	3.65	6.44			
					I264 (2)	3.76	5.83			
					**I290** (3)	3.72	21.76			
2	1N	**Y411** (1)	2.63	13.91	**N391** (5)	3.68	27.08	-8.20	1.03×10^6	0.016
					F403 (2)	3.74	4.12			
					L407 (2)	3.65	7.06			
					**V433** (3)	3.64	10.53			
					**L453** (6)	3.69	38.3			
	2N	**R410** (1)	3.09	13.72	**Y411** (7)	3.73	32.81	-7.90	6.21×10^5	0.016
					K414 (1)	3.80	7.87			
					**L430** (2)	3.74	39.15			
					**F488** (5)	3.65	28.64			
	8H	**Y411** (2)	2.84	16.14	**Y411** (2)	3.58	16.14	-8.35	1.32×10^6	0.010
					**R485** (3)	3.61	16.72			
					**F488** (12)	3.75	41.52			
