# Supplementary material for: Stereo-Selectivity of Human Serum Albumin to Enantiomeric and Isoelectronic Pollutants Dissected by Spectroscopy, Calorimetry and Bioinformatics
Source: PLoS One. 2011 Nov 2;6(11):e26186. doi: 10.1371/journal.pone.0026186 (PMC3206814; doi:10.1371/journal.pone.0026186)
Supplement: Table S2 — Interaction profile of pollutants with HSA after rigid docking with Autodock where number of contacts for corresponding residues to the ligand are given in parentheses and residues corresponding to ΔASA>10 Å2 are in bold. (PDF) [file pone.0026186.s004.pdf]

Table S2. Interaction profile of pollutants with HSA after ***rigid*** docking with AutoDock where number of contacts for corresponding residues to the ligand are given in parentheses and residues corresponding to ΔASA>10 Å^2 are in bold.

Site	Pollutants	H-Bonding			Hydrophobic interactions			ΔG	K_b	ΔC_P^{calc}
		Residues	Average distance(Å)	ΔASA (Å^2)	Residues	Average distance(Å)	ΔASA (Å^2)	(kcal. mol^{-1})	(M^{-1})	(kcal.mol^{-1}K^{-1})
	1N	Y150 (1)	2.79	5.79	**L238** (2)	3.45	26.95	-5.45	9.93×10^3	0.023
		**R257** (2)	2.94	13.48	**R257** (4)	3.59	13.48			
					**L260** (2)	3.87	11.29			
					A261 (3)	3.66	4.13			
					I264 (2)	3.58	8.41			
					**I290** (2)	3.50	13.02			
1	2N	**R257** (1)	2.66	12.75	**R257** (3)	3.69	12.75	-5.11	5.53×10^3	0.017
		A261 (1)	3.02	4.13	**L260** (5)	3.52	12.36			
		S287 (1)	3.24	4.65	A261 (1)	3.77	4.13			
					**I264** (2)	3.51	10.9			
					S287 (3)	3.56	4.65			
					**I290** (2)	3.84	13.62			
					A291 (4)	3.57	7.64			
	8H	Y150 (1)	2.72	5.89	**L238** (2)	3.80	25.27	-5.30	7.63×10^3	0.036
		**R257** (2)	3.14	13.47	**R257** (1)	3.18	13.47			
					**L260** (1)	3.81	10.41			
					A261 (1)	3.73	4.13			
					I264 (1)	3.59	7.19			
					**I290** (3)	3.66	13.53			
					**A291** (3)	3.73	23.5			
	1N	**Y411** (1)	2.83	10.75	L387 (4)	3.30	5.53	-5.17	6.17×10^3	0.017
					I388 (1)	3.82	6.27			
					**N391** (9)	3.56	17.35			
					L407 (4)	3.55	7.58			
					**Y411** (1)	3.82	10.75			
					L430 (3)	3.55	6.44			
					V433 (3)	3.36	9.62			
					**L453** (7)	3.67	18.85			
2	2N	R485 (1)	3.32	8.36	L387 (2)	3.33	5.53	-5.46	1.00×10^4	0.012
					**N391** (4)	3.48	14.24			
					L407 (2)	3.51	7.58			
					**Y411** (1)	3.89	15.5			
					L430 (6)	3.65	9.75			
					V433 (2)	3.36	6.01			
					**L453** (7)	3.60	21.45			
					R485 (1)	3.72	8.36			
	8H	L430 (1)	2.96	6.56	L387 (4)	3.50	5.53	-4.79	3.25×10^3	0.020
					I388 (2)	3.67	6.76			
					**N391** (7)	3.60	17.81			
					L407 (1)	3.90	7.58			
					L430 (1)	3.77	6.56			
					**V433** (2)	3.64	10.86			
					**L453** (5)	3.54	18.81			
